# The Impact of a Pharmacist-Driven *Staphylococcus aureus* Bacteremia Initiative in a Community Hospital: A Retrospective Cohort Analysis

**DOI:** 10.3390/pharmacy9040191

**Published:** 2021-11-25

**Authors:** Nate J. Berger, Michael E. Wright, Jonathon D. Pouliot, Montgomery W. Green, Deborah K. Armstrong

**Affiliations:** 1 Department of Pharmacy, Williamson Medical Center, Franklin, TN 37067, USA; miwright@wmed.org (M.E.W.); jpouliot@wmed.org (J.D.P.); Montgomery.green@belmont.edu (M.W.G.); debi.armstrong@comcast.net (D.K.A.); 2 Department of Pharmacy, Methodist Health System, Dallas, TX 75203, USA; 3 Department of Pharmacy Practice, College of Pharmacy and Health Sciences, Lipscomb University, Nashville, TN 37204, USA; 4 Department of Pharmacy Practice, College of Pharmacy, Belmont University, Nashville, TN 37212, USA

**Keywords:** *Staphylococcus aureus*, bacteremia, bloodstream infection, pharmacist

## Abstract

Purpose: *Staphylococcus aureus* is a leading cause of bacteremia with a 30-day mortality of 20%. This study evaluated outcomes after implementation of a pharmacist-driven *Staphylococcus aureus* bacteremia (SAB) initiative in a community hospital. Methods: This retrospective cohort analysis compared patients admitted with SAB between May 2015 and April 2018 (intervention group) to those admitted between May 2012 and April 2015 (historical control group). Pharmacists were notified of and responded to blood cultures positive for Staphylococcus aureus by contacting provider(s) with a bundle of recommendations. Components of the SAB bundle included prompt source control, selection of appropriate intravenous antibiotics, appropriate duration of therapy, repeat blood cultures, echocardiography, and infectious diseases consult. Demographics (age, gender, and race) were collected at baseline. Primary outcome was in-hospital mortality. Compliance with bundle components was also assessed. Results: Eighty-three patients in the control group and 110 patients in the intervention group were included in this study. Demographics were similar at baseline. In-hospital mortality was lower in the intervention group (3.6% vs. 15.7%; *p* = 0.0033). Bundle compliance was greater in the intervention group (69.1% vs. 39.8%; *p* < 0.0001). Conclusions: We observed a significant reduction in in-hospital mortality and increased treatment bundle compliance in the intervention cohort with implementation of a pharmacist-driven SAB initiative. Pharmacists’ participation in the care of SAB patients in the form of recommending adherence to treatment bundle components drastically improved clinical outcomes. Widespread adoption and implementation of similar practice models at other institutions may reduce in-hospital mortality for this relatively common and life-threatening infection.

## 1. Introduction

*Staphylococcus aureus* is a leading cause of community- and hospital-acquired bacteremia. In patients with *Staphylococcus aureus* bacteremia (SAB), 30-day all-cause mortality is approximately 20% [1,2,3]. Furthermore, risk of mortality over one year has been demonstrated to be 44.6% [4]. Treatment failure (i.e., death within 30 days, persistent bacteremia longer than seven days while receiving therapy, bacteremia recurrence within 30 days, or in-hospital mortality) is common in patients with SAB [1]. Guidelines published by the Infectious Diseases Society of America make specific recommendations for the management of SAB [5] to reduce the risk of complications and relapse. These recommended interventions have been referred to as quality-of-care indicators (QCIs) [2,6,7,8] or quality performance measures [9] in the literature, but adherence to these measures is variable [2,6,7,8,9,10,11,12]. Quality improvement programs for the management of SAB are recommended [3] and many hospitals have implemented initiatives seeking to standardize care for SAB with protocols for a treatment bundle consisting of several QCIs [3,6,7,8,9,10,11,13,14]. Several studies have not only demonstrated improved adherence to QCIs, but that adhering to a bundle-based treatment approach is associated with improved prognosis [6,7,8].

Antibiotic stewardship programs (ASPs) are designed to improve patient care by recommending interventions to improve antibiotic use and clinical outcomes [14]. Furthermore, the American Society of Health-System Pharmacists states that responsibilities of pharmacists for antimicrobial stewardship include the promotion of optimal use of antimicrobials and to provide education and information about antimicrobial stewardship to health professionals and patients [15]. Interventions performed by ASP pharmacists have previously demonstrated reductions in 30-day mortality for methicillin-resistant *Staphylococcus aureus* (MRSA) bacteremia [8]. Although many studies evaluating the impact of ASP pharmacist interventions focus on process outcomes such as adherence to guidelines and reduction in antibiotic usage and associated costs [14], our study sought to evaluate the pharmacist’s interventions on clinical outcomes, including in-hospital mortality and 30-day mortality. ASP pharmacists are well-positioned to respond to blood cultures positive for *Staphylococcus aureus* and make timely recommendations to the patient-care team for the appropriate management of SAB. Recommendations made by ASP pharmacists include initiation of, or de-escalation to appropriate therapy, adequate duration of therapy, repeat blood cultures, performing echocardiogram, and obtaining consultation with infectious disease specialist. Previous studies examining ASP initiatives aimed at improving SAB outcomes by incorporating QCIs into a standardized treatment bundle have demonstrated improved adherence to QCIs [9,10,11]. Although, these studies demonstrated reductions in clinical outcomes, such as 30-day mortality and readmissions, these reductions did not meet statistical significance [9,10,11]. Our study differs in that our primary outcome of interest was in-hospital mortality and not adherence to QCIs.

The management of SAB is the responsibility of the primary hospital team at our institution, a medium-sized community hospital located in middle Tennessee, USA. Prior to 2015, no formalized institutional treatment guidelines existed to provide support or direct treating physicians to adhere to evidence based QCIs. As a result, practice varied by provider. Our institution’s ASP sought to improve the quality of care for SAB by implementing a pharmacist-driven treatment bundle and follow-up to ensure optimal management of this high-risk infection. The purpose of this study was to evaluate how a standardized, pharmacist-driven, ASP treatment bundle for SAB impacted clinical outcomes and adherence to QCIs in a community hospital.

## 2. Methods

### 2.1. Study Design

A retrospective historical control study was conducted evaluating the impact of a pharmacist-driven treatment algorithm for SAB at a 185-bed community, non-academic hospital located in Tennessee, USA. The pre-intervention (control) group and intervention group included patients admitted between 1 May 2012 and 30 April 2015 and 1 May 2015 and 30 April 2018, respectively. Approval of waiver of authorization from a third-party institutional review board (Sterling IRB, IRB ID: 7567-NBerger, 9 October 2019) was obtained.

All patients admitted with one or more positive blood cultures for *Staphylococcus aureus* during the study periods ≥18 years of age were eligible. Positive blood cultures were detected through review of microbiological records and the date of hospital admission was considered the index date to determine group assignment. Patients were excluded for any of the following: pregnancy at time of positive blood culture and/or during treatment period, prisoners, coinfection with methicillin-resistant Staphylococcus aureus (MRSA) and methicillin-susceptible Staphylococcus aureus (MSSA), patients transferred to outside hospital, patients who are on hospice or comfort care or who were referred to hospice or comfort care within 24 h of admission, patients who expired before receipt of positive blood cultures, patients with documented SAB infection prior to index admission, or patients whose blood cultures were collected from the emergency department that were not admitted within 72 h.

### 2.2. SAB Treatment Algorithm

The SAB initiative was approved by the hospital ASP and began in April 2015. This initiative consisted of a SAB treatment algorithm of evidence-based and guideline recommended bundle components, as well as pharmacist response and follow-up to ensure bundle compliance. Pharmacists were notified via email of blood cultures positive for *Staphylococcus aureus* and responded during business hours (08:00 to 17:00), seven days per week. Pharmacists contacted the provider directly via telephone or during multidisciplinary care team rounding, if needed, with evidence-based suggestions for antimicrobial therapy, monitoring, and recommendation for infectious disease (ID) consult. The components of the SAB bundle were source control, treatment with appropriate intravenous antibiotics, duration of treatment ≥14 days for uncomplicated bacteremia or ≥28 days for complicated bacteremia, repeat blood cultures within 2–4 days after initiation of anti-staphylococcal antibiotics, echocardiography, and ID consult.

### 2.3. Data Collection

Data were obtained from a review of the electronic medical record (EMR) and recorded into a uniform de-identified data collection form. Collected data included demographic information, comorbidities, dates of admission/discharge, date/time of blood culture collection, date/time of blood culture result, microbiological results, date/time of death, antibiotic treatment selected, date/time of initial administration of definitive therapy, date/time of defervescence, and whether treatment bundle components were performed.

### 2.4. Variables and Definitions

The primary endpoint was all-cause in-hospital mortality. Secondary clinical endpoints included seven-day mortality, 30-day mortality, length of hospital stay, time to defervescence, time to definitive therapy, and duration of antibiotics. Seven- and 30-day mortality rates were right censored if data was incomplete. For patients with no follow-up data in the EMR at 30 days, a search of public records was performed to identify dates of death outside of the hospital. Data for patients without either a follow-up in the EMR or no identifiable date of death in the public record search were considered missing. Length of hospital stay was defined as the number of days from admission to discharge. Time to defervescence was determined as the time from collection of first positive blood culture until resolution of fever (<100.4 °F) and no subsequent fever over the next 48 h. Definitive therapy was defined as the antibiotic treatment selected during hospitalization to be continued for the remainder of the treatment duration. Time to definitive therapy was determined as the time from the result of the first positive blood culture to administration of definitive therapy. Length of treatment duration was considered as the time from the first negative blood culture and included planned outpatient treatment. Compliance with individual treatment algorithm bundle components were assessed. Overall compliance was considered achieved if all of the individual treatment algorithm bundle components met criteria for compliance. Appropriate definitive therapy was defined as cefazolin, nafcillin, oxacillin, or ampicillin-sulbactam for MSSA or vancomycin, daptomycin, or linezolid for MRSA. Source control was considered appropriate if source of infection was identified and removal or drainage was achieved if possible. Susceptibilities of *S.* *aureus* were based on Clinical Laboratory Standards Institute guidelines. Charlson Comorbidity Index was calculated for each patient and used to assess severity of chronic disease states. Uncomplicated bacteremia was defined as exclusion of endocarditis, metastatic infection, or deep tissue infection [15].

### 2.5. Statistical Analysis

Descriptive statistics were utilized to compare baseline characteristics of each group. Outcomes were evaluated using t-test for continuous variables, Wilcoxon Rank test for ordinal variables, and chi-squared test for categorical variables. A *p*-value of <0.05 was considered statistically significant assuming two-tailed analysis. A post hoc power analysis was planned to assess power given that the study was limited in sample size by time frames of SAB implementation. All statistical analysis was completed by study authors utilizing JMP Pro version 14.3.0 (SAS Institute Inc., Cary, NC, USA).

## 3. Results

**Figure 1:**
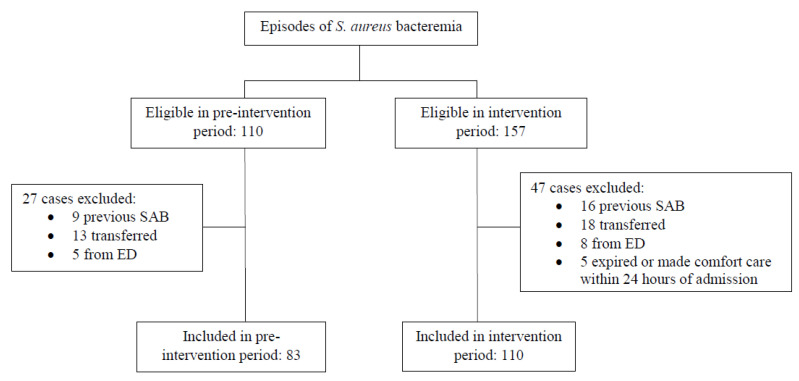
Flow chart of patients included in the study. Abbreviation: SAB, *Staphylococcus aureus* bacteremia; ED, emergency department.

During the study period, 267 patients with SAB were identified, of which 193 met inclusion criteria, including 110 in the intervention group and 83 in the control group (Figure 1). Baseline demographics were similar between groups and are shown in Table 1.

Clinical outcomes and bundle compliance outcomes are displayed in Table 2. The primary endpoint of in-hospital mortality was significantly lower in the intervention group compared to the control group (4/110 [3.6%] vs. 13/83 [15.7%], *p* = 0.0033). The absolute risk reduction of 12.1% observed in in-hospital mortality corresponds to a number needed to treat of nine. Secondary endpoints for the intervention group were also significantly lower for both seven-day mortality (4/110 [3.6%] vs. 10/83 [12.1%], *p* = 0.026) and thirty-day mortality (8/110 [7.3%] vs. 17/83 [20.5%], *p* = 0.007). Mean length of stay was similar between groups (10.6 days for intervention group vs. 10.2 for control group, *p* = 0.72). Mean duration of antibiotics also did not differ between groups (28.3 days vs. 27.0 days, *p* = 0.57). Time to definitive therapy was significantly shorter in the intervention group (11.7 h vs. 24.5 h, *p* = 0.0011). No significant difference was detected for time to defervescence (31.5 h vs. 40.9 h, *p* = 0.49).

The intervention group was associated with increased adherence to the entire bundle compared to the control group (76/110 [69.1%] vs. 33/83 [39.8%], *p* < 0.001). Statistically significant differences in adherence favoring the intervention group were seen for all the individual treatment algorithm bundle components, except for ordering of an echocardiogram (Table 2).

Definitive therapy selected is displayed in Table 3. During the pre-intervention period vancomycin was selected as definitive therapy 53.0% of the time (44/83), while vancomycin was selected 36.4% of the time (40/110) during the intervention period. While MRSA infections occurred 43.4% (36/83) and 40.9% (45/110) of the time during the control and intervention periods, respectively. Cefazolin was the most frequently selected definitive therapy during the intervention period with 41.8% (46/110) of patients receiving cefazolin, compared to only 7.2% (6/83) during the control period.

## 4. Discussion

The results of this study show that the pharmacist-driven ASP SAB bundle initiative was associated with reduced mortality and improved adherence to QCIs. This initiative implemented in our hospital consisted of real-time pharmacist notification from the microbiology lab, pharmacist review and intervention to support adherence to quality-of-care indicators, and follow-up to ensure continued best practices during admission and for discharge. Previous studies examining ASP-pharmacist driven initiatives have shown increased adherence to QCIs but have failed to demonstrate a statistically significant mortality benefit [9,10,11]. This study adds to the existing literature by showing that a pharmacist-driven ASP standardized treatment bundle for the management of SAB may improve mortality rates.

Our study differs from previous research focusing on pharmacist-driven management of SAB in that our primary outcome was clinical in nature, in-hospital mortality. Other published studies that have evaluated the impact of pharmacist-driven ASP treatment bundles for SAB focused on bundle adherence as the primary outcome (9-11). Nguyen et al. evaluated the impact of an ASP-driven SAB treatment bundle in which pharmacists were alerted to positive cultures for SAB and provided standardized verbal recommendations [9]. Implementation of this pharmacist-driven treatment bundle was associated with improved adherence to the complete treatment bundle (56.1% pre-intervention vs. 84.1% intervention, *p* < 0.001), however, failed to demonstrate statistically significant reduction in 30-day mortality (19.5% pre-intervention vs. 11.4% post-intervention, *p* = 0.2) [9]. The 30-day mortality rates demonstrated by Nguyen et al. were similar to mortality rates demonstrated within our study for both the pre-intervention (19.5% vs. 20.5%) and post-intervention periods (11.4% vs. 7.3%). Another study by Wenzler et al. which evaluated a pharmacist-driven automated intervention for SAB, similarly, found significantly greater adherence to the bundle of QCIs after implementation (68.9% pre-intervention vs. 92.3% intervention, *p* = 0.008) [11]. Although this study also failed to show significant reduction in 30-day all-cause mortality (15.6% pre-intervention vs. 2.6% intervention, *p* = 0.063) [11]. The 30-day mortality risk reduction demonstrated by Wenzler et al. was similar to the risk reduction demonstrated between our cohorts (13% vs. 13.2%) [11]. A third study by Remtulla et al. assessed the impact of unsolicited, standardized, SAB interventions delivered by ASP pharmacists on bundle adherence and outcomes [10]. Similarly, to the others, this study demonstrated improved adherence to the SAB bundle (29.0% pre-intervention vs. 72.8% intervention, *p* < 0.001) but did not demonstrate a significant reduction in in-hospital (27.4% pre-intervention vs. 21.2% intervention, *p* = 0.33) or 30-day mortality rates (29.0% pre-intervention vs. 21.9% intervention, *p* = 0.28) [10]. The authors noted that this study was underpowered to detect a mortality benefit [10]. The results of all three previous pharmacist-driven intervention studies demonstrated a 30-day mortality risk reduction that did not meet statistical significance. Comparatively, our study may have been adequately powered to detect a difference in mortality.

One of the elements of SAB management that has been most studied and strongly supported in the literature is consultation with an ID specialist. Previous work has demonstrated an association between ID consultation and decreased mortality, as well as improved adherence to QCIs [2,16]. In this study, infectious disease consultation was improved after SAB bundle implementation, but rate of ID consultation was high at baseline, occurring in 88.0% of patients, pre-intervention. The high rate of ID consultation in both groups suggests that pharmacist-driven recommendations for the SAB bundle may improve outcomes and adherence to the SAB bundle independent of ID consultation.

Selection of appropriate definitive therapy was increased in the intervention group compared to the control. It appears that a significant driver of the mortality difference detected may have been the improved use of beta-lactam antibiotics, specifically cefazolin, for the treatment of MSSA. Previous research has demonstrated that the use of beta-lactam antibiotics for the definitive treatment of MSSA is associated with reduced mortality compared to vancomycin [17]. The proportion of MRSA isolates was similar between both study groups, with MRSA accounting for 42% of infections. Furthermore, the proportion of MRSA isolates is consistent with previous studies conducted within the United States [9,18]. Appropriate definitive therapy was selected more frequently in the intervention group, and time to definitive therapy was shorter (11.7 h vs. 24.5 h, *p* = 0.001). Increased rates of appropriate definitive therapy as well as the shorter times to definitive therapy seen with a pharmacist intervention and follow up in our study may be important factors contributing to the mortality difference detected between groups.

One potential confounding factor that occurred during the intervention period was the implementation of a rapid polymerase chain reaction (rPCR) assay for blood culture identification in October 2015. Prior to implementation of rPCR assay, pathogen identification for gram positive cocci took 24–48 h from the time of positive blood culture, whereas this could be completed in less than 2 h with rPCR. Faster identification of *S. aureus* and the presence or absence of methicillin resistance may have contributed to the improved outcomes seen in our study. However, previous literature evaluating the clinical impact of implementation of rapid blood culture identification diagnostics have failed to demonstrate a difference in mortality. Bauer et al. evaluated the impact of implementation of rPCR along with treatment recommendations by an ID pharmacist on clinical outcomes for MRSA/MSSA. Despite faster initiation of appropriate therapy, no difference in in-hospital mortality between pre- and post-rPCR groups was detected (26% vs. 18%; odds ratio, 0.65; 95% confidence interval, 0.30–1.39) [19]. Another study by Frye et al. compared outcomes before and after implementation of rPCR for identification of *S. aureus* without any intervention or ASP follow-up and found no difference in time to optimal antibiotic therapy for MRSA or MSSA. Additionally, no difference in in-hospital mortality was found between groups (12.7% vs. 12.7%; *p* > 0.1). The authors concluded that although implementation of rPCR has the potential to improve antibiotic use, it should be utilized along with additional intervention and process optimization to facilitate use of the rPCR result [20]. Other studies evaluating the clinical impact of rPCR implementation for bacteremia have similarly failed to demonstrate a mortality benefit [21,22,23]. While rPCR for *S. aureus* may shorten the times to identification and de-escalation of therapy, current evidence has failed to demonstrate an association with a reduction in mortality. Within our study population, all 20 patients within the intervention cohort, prior to implementation of rPCR, were alive at hospital discharge and 7 days, with 1/19 (5.3%) patients deceased at 30 days. For comparison, 4/90 (4.4%) of patients within the intervention cohort after the implementation of rPCR met the primary endpoint for in-hospital mortality, with 4/83 (4.8%) deceased at 7 days, and 7/75 (9.3%) at 30 days. Based on this comparison of mortality rates within the intervention group, for periods pre- and post-implementation of rPCR, rPCR testing did not improve mortality. These results along with our own suggest that, regardless of implementation of rPCR, pharmacist review and adherence to the SAB treatment bundle are important drivers in the reduction in mortality rates seen in this study.

Overall adherence to the SAB bundle increased from 39.8% to 69.1% after implementation (*p* < 0.001). Significant increases were seen for all seven measured bundle components except echocardiography (*p* = 0.1). Our results are consistent with previous literature that implementation of a pharmacist-driven ASP SAB bundle improves adherence to important QCIs for the optimal management of SAB [9,10,11].

This study took place in a community hospital with 185 beds. Much of the literature detailing implementation of ASP SAB bundles is generated from academic medical centers [9,10,11]. The results of this study demonstrate that the implementation of an ASP SAB bundle in a community hospital is both practical and associated with improved outcomes. The type of intervention described here is both comparable in scope and scale to initiatives from larger hospitals and this study demonstrates that similar interventions may be effectively incorporated into any size hospital with a functioning ASP. SAB is a common infection, therefore, it is important for hospitals of all sizes to implement quality improvement processes, such as a pharmacist-driven SAB bundle, in an effort to improve clinical outcomes.

This study does have limitations to consider. The retrospective nature of this study design does not allow causality to be assigned to the intervention or any of its components. Additionally, there may be differences between the populations that were not captured as part of the data collection that may have confounded the results. Furthermore, the influence of potential differences in standards of practice over time is not measured when comparing the intervention and control groups. Patients were not formally followed after hospital discharge and therefore some data was missing. More patients were lost to follow-up in the intervention group for seven-day mortality (7 patients vs. 2 patients) and 30-day mortality (11 patients vs. 3 patients). However, statistical analysis was performed for seven- and 30-day mortality using both intention-to-treat and per protocol populations (which excluded patients lost to follow-up) and the results remained significant in both analyses.

In conclusion, this study supports the role of the pharmacist in the management of SAB through an ASP implemented treatment bundle to improve clinical outcomes and adherence to evidence-based treatment recommendations. Through a prospective pharmacist review, recommendations made to the multidisciplinary care team, and follow-up, associations with reduced mortality and increased adherence to evidence-based quality care measures for the treatment of SAB were demonstrated.

## Figures and Tables

**Table 1:** Demographics.

Characteristic	All Patients (*n* = 193)	Intervention (*n* = 110)	Control (*n* = 83)	*p* ^a^
Mean age, years	66.5	65.1	68.3	0.17
Male	111 (57.5)	59 (53.6)	52 (62.7)	0.21
Caucasian	173 (89.6)	100 (90.9)	73 (88.0)	0.66
African American	17 (8.8)	9 (8.2)	8 (9.6)	
Native Hawaiian/Pacific Islander	3 (1.6)	1 (0.9)	2 (2.4)	
MSSA	112 (58.0)	65 (59.1)	47 (56.6)	0.73
MRSA	81 (42.0)	45 (40.9)	36 (43.4)	
Mean Charlson Comorbidity Index	4.04	4.02	4.06	0.76

Data are presented as No. of patients (%) unless specified otherwise. Abbreviations: MSSA, methicillin-sensitive *Staphylococcus aureus*; MRSA, methicillin-resistant *Staphylococcus* aureus. ^a^ *p* for intervention group vs. control group.

**Table 2:** Outcomes.

Outcomes	All Patients (*n* = 193)	Intervention (*n* = 110)	Control (*n* = 83)	*p* ^a^
Clinical outcomes				
In-hospital mortality	17 (8.8)	4 (3.6)	13 (15.7)	0.0033
Seven-day mortality	14 (7.3)	4 (3.6)	10 (12.1)	0.026
Thirty-day mortality	25 (13.0)	8 (7.3)	17 (20.5)	0.007
Mean length of stay (days)	10.4	10.6	10.2	0.72
Mean duration of antibiotics (days)	27.7	28.3	27.0	0.57
Time to definitive therapy (hours)	17.2	11.7	24.5	0.0011
Time to defervescence (hours)	35.5	31.5	40.9	0.49
Bundle compliance				
Overall compliance	109 (56.5)	76 (69.1)	33 (39.8)	<0.001
Infectious disease consult	181 (93.4)	108 (98.2)	73 (88.0)	0.003
Appropriate definitive therapy	161 (83.4)	104 (94.5)	57 (68.7)	<0.001
Source identified	154 (79.8)	94 (85.5)	60 (72.3)	0.025
Repeat blood cultures collected	181 (93.8)	110 (100)	71 (85.5)	<0.001
Echocardiogram performed	161 (83.4)	96 (87.3)	65 (85.5)	0.1
Appropriate duration of antibiotics	161 (83.4)	99 (90)	62 (74.7)	0.005

Data are presented as number of patients (%) unless specified otherwise. Time points refer to time of culture collection as baseline. ^a^ *p* is for intervention vs. control.

**Table 3:** Definitive Therapies.

Definitive Therapy Selected	All Patients (*n* = 193)	Intervention (*n* = 110)	Control (*n* = 83)
Cefazolin	52 (26.9)	46 (41.8)	6 (7.2)
Ceftriaxone	11 (5.7)	2 (1.8)	9 (10.8)
Cephalexin	1 (0.5)	1 (0.9)	0 (0)
Clindamycin	1 (0.5)	0 (0)	1 (1.2)
Daptomycin	10 (5.2)	7 (6.4)	3 (3.6)
Nafcillin	30 (15.5)	14 (12.7)	16 (19.3)
None	2 (1.0)	0 (0)	2 (2.4)
Sulfamethoxazole/trimethoprim	2 (1.0)	0 (0)	2 (2.4)
Vancomycin	84 (43.5)	40 (36.4)	44 (53.0)
Rifampin adjunct	13 (6.7)	6 (5.5)	7 (8.4)

Data are presented as number of patients (%).

## Data Availability

The data presented in this study are available on request from the corresponding author. The data are not publicly available due to privacy laws regarding protected health information.
